# Quantifying replicative senescence as a tumor suppressor pathway and a target for cancer therapy

**DOI:** 10.1038/srep17660

**Published:** 2015-12-09

**Authors:** Ignacio A. Rodriguez-Brenes, Dominik Wodarz, Natalia L. Komarova

**Affiliations:** 1 Department of Mathematics, Univeristy of California, Irvine, U.S.A; 2 Department of Ecology and Evolution, Univeristy of California, Irvine, U.S.A

## Abstract

To study quantitatively replicative senescence as a tumor suppressor mechanism, we investigate the distribution of a growing clonal cell population restricted by Hayflick’s limit. We find that in the biologically relevant range of parameters, if the imbalance between cell division and death is moderate or low (high death-to-birth ratio), senescence offers significant protection against cancer by halting abnormal cell proliferation at early pre-diagnostic stages of tumor development. We also find that by the time tumors are typically detected, there is a high probability that telomerase is activated, even if the cell of origin was telomerase negative. Hence, the fact that most cancers are positive for telomerase is not necessarily an indication that cancer originated in a telomerase positive cell. Finally, we discuss how the population dynamics of cells can determine the outcomes of anti-telomerase cancer therapies, and provide guidelines on how the model could potentially be applied to develop clinically useful tools to predict the response to treatment by telomerase inhibitors in individual patients.

Normal somatic cells are capable of only a limited number of divisions. This phenomenon known as replicative senescence, or Hayflick’s limit^1^, depends on the shortening of telomeres, which are repetitive sequences of DNA found at the end of linear chromosomes^2^. In cells that naturally lack telomere length-maintenance pathways, every time a cell divides telomere length decreases. If telomere length reaches a critically short size, senescence or apoptosis are triggered through DNA damage response pathways^3^. The rate of telomere attrition is affected by various factors including the levels of genotoxic stress in the cell and the presence of reactive oxygen species (ROS)^4^,^5^.

Replicative senescence might have evolved as a mechanism to protect against cancer in multicellular organisms^2^. Cancer is driven by the clonal expansion of cells, which have accumulated a number of mutations that allow them to proliferate in an uncontrolled manner^6^. Conceptually, by limiting the number of times cells may divide, replication limits would cap the size of a clonal cell population preventing an incipient growth from reaching a potentially harmful size. Furthermore, since mutations typically occur during cell division, limiting the possible number of divisions reduces the probability of acquiring multiple mutations, which according to the multi-hit theory of carcinogenesis is necessary for the full progression towards malignancy^6^.

A large number of mathematical models have been developed that explore different aspects of telomere dynamics and replicative senescence. These models use a wide range of approaches from population dynamics (see e.g.^7^,^8^,^9^) to detail descriptions of telomere function^10^,^11^,^12^,^13^,^14^. More specifically, the connection between replicative limits and cancer has been studied mathematically from various angles, including their effects on stem-cell-driven tumors^15^,^16^, their relation to tissue architecture and cancer risk^17^,^18^, and their influence on precancerous mutations^19^. In spite of these growing modeling efforts, several fundamental aspects of the relationship between replicative senescence and cancer remain poorly understood.

In this article we investigate quantitatively how effective replicative limits really are as a tumor suppressor pathway, once abnormal cell growth has begun. Hence, we only focus on cell populations that are affected by replicative limits. And, although other factors (endogenous or environmental) can halt cell proliferation^20^, the focus of this article is on replicative senescence and its effects on population dynamics. To address this question we constructed a stochastic model of cell proliferation based on two basic biological parameters: the replication capacity of the originating cell and the ratio between the rates of cell death and cell division. We then focus our attention on the distribution of the maximum cell populations, and on the probability of escaping replicative limits through telomerase activation. Telomerase, which is expressed by the majority of cancer cells^21^, is an enzyme that extends telomere length allowing cells that express it at sufficient levels to bypass Hayflick’s limit^2^. Finally, we discuss applications of the theory to assess the effectiveness of anti-telomerase cancer therapies in individual patients, based solely on canonical telomerase activity inhibition.

We find that based on the distribution of the maximum population sizes, replicative limits offer significant protection against cancer if the imbalance between cell division and death is moderate or low (that is, when dynamics are characterized by a high death-to-birth ratio). Moreover, our analysis suggests that on a significant number of occasions, replicative limits might halt abnormal growth before the tumor progresses beyond the microscopic level. Hence, replicative senescence might act as a tumor suppressor before most abnormalities are ever detected.

We also found that the probability of escaping replicative limits through a mutation that activates telomerase considerably increases the risk posed by abnormal cell proliferation. In particular, we examined whether tumors must originate in cells that are already telomerase positive (such as stem cells), or whether telomerase can be acquired later, after uncontrolled proliferation begins. We found that by the time tumors reach a size where they are typically detected, there is a high probability that telomerase is activated in the cell population, even if the cell of origin was telomerase negative. Thus, the fact that most tumors are telomerase positive by the time they are diagnosed is not necessarily an indication that they originated from a telomerase positive cell.

Finally, we discuss how the population dynamics of cells restricted by replicative limits can ultimately determine the success of telomerase inhibitor therapies in individual patients. We propose how the rates of cell division and death, and the distribution of telomere length in cancer cells could be potentially used to develop quantitative tools to predict the probability of success of anti-telomerase therapies in individual patients; and how these odds might improve when anti-telomerase strategies are combined with other forms of treatment.

## Results

### Mathematical framework

**Figure 1:**
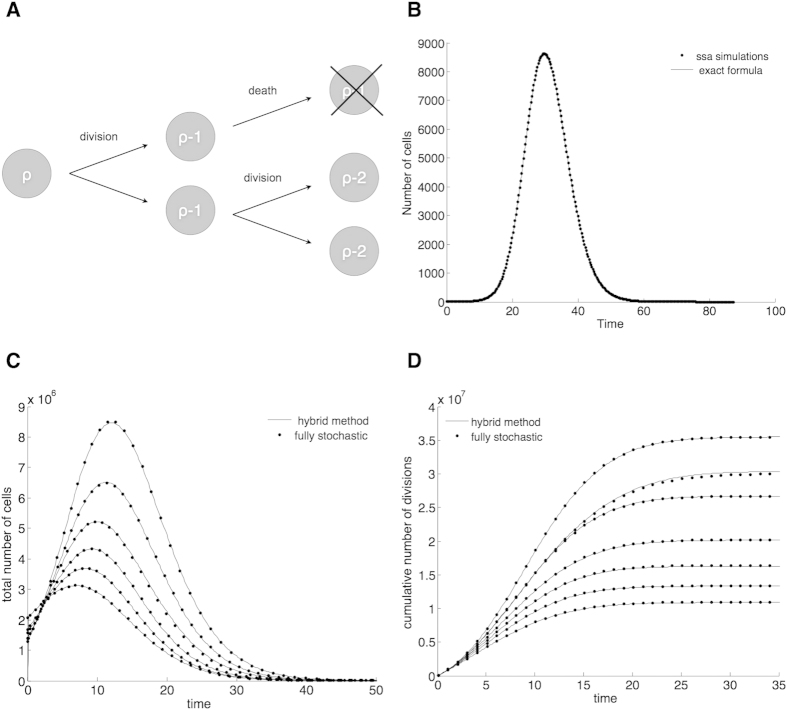
(**A**) Cells have a limited replication capacity equal to the maximum number of times they may divide. A cell with replication capacity *ρ* > 0 can either divide with probability *q* or die with probability 1 − *q*. If division occurs the replication capacity of each daughter cell decreases by one. (**B**) Expected number of cells vs time. At time *t* = 0 there is one single cell with replication capacity *ρ* = 30 and division probability *q* = 0.7. The solid line is calculated using Eq. 7. Dots are the average of 1000 independent random simulations. (**C**,**D**) Simulations of the total number of cells (**C**) and the cumulative number of divisions (**D**) calculated using the hybrid algorithm (solid lines) and the fully stochastic algorithm (dots). In the hybrid algorithm the switch between stochastic and deterministic phases takes place when the number of proliferating cells equals 10^6^. Panel (**C**) plots the trajectories after the stochastic-deterministic transition takes place (x-axis indicates time elapsed after the transition). Panel (**D**) plots the whole span of the simulations. The parameters (*k* = 40, *q* = 0.75) are such that the implementation of the fully stochastic algorithm through the entire lifetime of the population is computationally feasible. See text for discussion.

To model Hayflick’s limit we assume that every cell has a limited replication capacity *ρ* equal to the maximum number of times it may divide. We also assume that the replication capacities of daughter cells are equal. Given these assumptions, in the model when a cell with replication capacity *ρ* > 0 divides, it produces two cells, each with replication capacity *ρ* − 1 (Fig. 1A). When a cell’s replication capacity is exhausted (*ρ* = 0), the cell becomes senescent and stops dividing. This is a common scheme to model Hayflick’s limit at the level of cell populations^9^,^15^,^19^.

Our replication capacity model is consistent with any linear or non-linear deterministic model of telomere shortening, as long as the replication capacity of daughter cells are equal. In general the replication capacities of sister cells can vary stochastically; however, when dealing with cell populations across multiple generations these potential differences average out. There are multiple detailed stochastic models of telomere shortening at the cellular level^4^,^10^,^11^,^14^. The drawback with these models is that they are unable to simulate populations much greater than 10^4^ cells. To deal with this difficulty, we developed a novel hybrid stochastic-deterministic approach, which allows us to systematically study the population dynamics of cells restricted by replicative limits, across population sizes that are biologically relevant and which span many orders of magnitude.

It is also important to clarify that we do not track or model telomere length. The dynamics of telomere shortening at the cellular level are complex and non-linear^14^. In particular, cell division potential is not linearly dictated by telomere length. In general the processes that contribute to telomere shortening and the induction of senescence depend on multiple factors such as tumor type, the cells’ stress levels^4^, and the cells’ microenvironment^5^.

We are interested in the dynamics of a cell population that is growing without control; hence, we assume that cells die and divide at constant rates *a*_div_ and *a*_die_, with *a*_div_ > *a*_die_. If *X*_*ρ*_(*t*) is the average number of cells with replication capacity *ρ* at time *t* and *k* is the maximum replication capacity, then the dynamics are described by the ODE system:


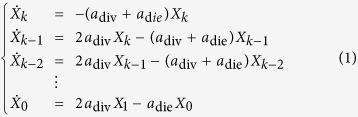


Cancer is thought to originate from a single founder cell^6^. When the number of cells is very small, stochastic fluctuations can play a fundamental role. We thus consider a probabilistic formulation. Let us define


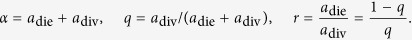


Assume that the times for cell division and death are exponentially distributed with means 1/*a*_div_ and 1/*a*_die_. Then at an exponentially distributed time with mean 1/*α* a cell with replication capacity *ρ* > 0 will either divide with probability *q* or die with probability 1 − *q*. Cell death in a senescent cell can be modeled directly as a stochastic event with rate *a*_die_; however, it will be useful to think about the following equivalent formulation: at an exponentially distributed time with mean 1/*α* a cell with replication capacity *ρ* = 0 will either die with probability 1 − *q* or simply remain in the population. With this choice of exponentially distributed times for cell division and death the expected number of cells in the stochastic model coincides with the solution of Eq. 1. For large populations however, simulations with different distributions, but the same means (1/*a*_div_ and 1/*a*_die_), can also provide good agreement with the deterministic model. In the discussion section we outline how different distributions can be implemented.

### Expected number of cells

The expected number of cells can be calculated using induction and integration by parts to solve Eq. 1, or using probability methods. Since our focus for the rest of the paper will be on the stochastic model, we derived the solution using probability ideas found in^8^. Let *k* be the maximum replication capacity and 
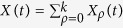
 the total expected number of cells at time *t*. Assume that there is a single cell with replication capacity *ρ* = *k* at time *t* = 0 and call 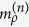 the expected number of cells with replication capacity *ρ* in generation *n*. We have:


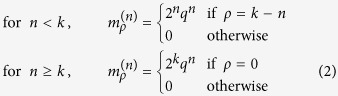


A cell in generation *n* is the result of *n* events that occur at exponentially distributed times. The time and number of these events is then a Poisson process with intensity *α*. Hence, the probability that a cell at generation *n* is born before time *t* equals:


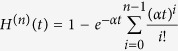


The probability that a cell of generation *n* is present at time *t* is the same as the probability that the cell was born before time *t*, but has not divided by time *t*, which equals *H*^(*n*)^(*t*) − *H*^(*n*+1)^(*t*). If *ρ* > 0, from Eq. 2 every cell with replication capacity *ρ* must belong to generation *k* − *ρ* and thus 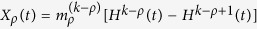. For *ρ* > 0 we have then:


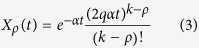


From Eq. 2 we can also obtain an expression for the expected number of senescent cells by summing over all generations *n* ≥ *k*. We have 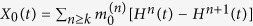 and hence:


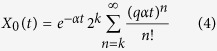


The expected number of cells at time *t* arising from a single cell with replication capacity *k* at *t* = 0 is then equal to


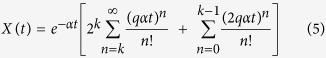


Fig 1B plots this quantity using the analytical solution and the average of independent random simulations.

We can generalize Eq. 5 in two ways. First, if senescent cells die at a different rate 1 − *p* ≠ 1 − *q*, then for *n* ≥ *k* we now have 
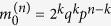
. Second, we can include different initial conditions. If *k* is now the maximum replication capacity found in the population at *t* = 0, we have:


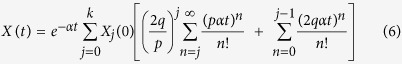


From here on we focus on populations arising from a single founder cell and for simplicity assume *p* = *q*. Moreover, we can express the system in terms of dimensionless units of time by making *α* = 1. The model then depends on two basic parameters, *k* and *q*. We also note that for *k* large the factorials in Eq. 5 are difficult to evaluate in standard floating point arithmetic. It is then useful to have an alternative representation in terms of special functions. Let 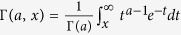 be the normalized incomplete upper Gamma function and *γ*(*a*, *x*) the normalized incomplete lower Gamma function, then using standard identities we can rewrite Eq. 5 as:


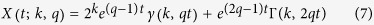


Without replication limits, if the division rate is greater than the death rate (*q* > 0.5), the expected cell population grows without bound as *e*^(2*q*−1)*t*^. In contrast, with replication limits the maximum size of the population is clearly capped; thus, one fundamental question is, what is the maximum size of the expected cell population?

**Figure 2:**
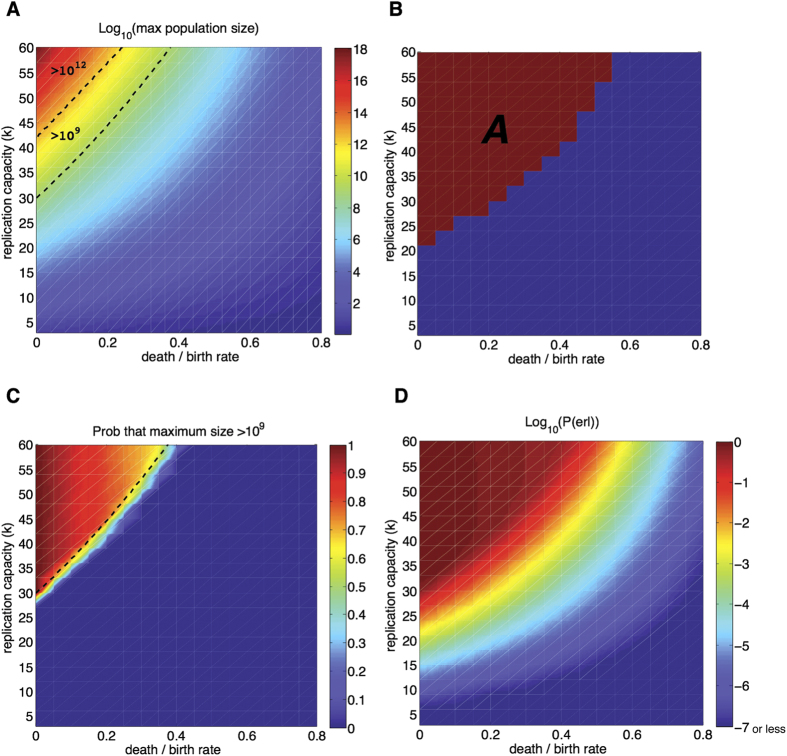
(**A**)Maximum size of the expected cell population 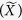 as a function of the replication capacity (*k*) and the death-to-birth ratio (r) of the originating cell. Dashed lines indicate the regions where the maximum population is greater than 10^9^ cells (detection threshold) and 10^12^ cells (lethal burden). See text for discussion. (**B**) Region *A* identifies parameters for which the expected maximum population size is greater than 10^6^. (**C**) Heat map: Probability that the maximum population size is greater than 10^9^. Dashed line: region where the maximum size of the expected cell population, 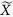, is greater than 10^9^. (**D**) Heat map of the probability of escaping replication limits as a function of the replication capacity of the originating cell and the death-to-birth ratio. The value of P(erl) is estimated as the midpoint between the upper and lower bounds in Eq. 9. Results based on 10^4^ simulations per point.

Figure 2A depicts the maximum size of the expected cell population, hereafter called 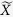 and computed numerically from Eq. 7, as a function of *k* and the death-to-birth ratio *r* = (1 − *q*)/*q*. The upper bound of *k* reflects the experimentally observed average number of divisions in human embryonic fibroblasts, which on average divide between 40–60 times in cell culture^1^. The replication capacity for cells from older donors is lower and decreases with age^22^. The death-to-birth ratio (*r*) is a measurement of the deviation from homeostatic conditions. In normal tissue *r* = 1, abnormal growth occurs when *r* < 1.

We delimit two regions in Fig. 2A indicating where 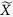 is greater than 10^9^ and 10^12^ cells. The 10^12^ threshold is an order of magnitude in which several types of cancers (e.g. breast cancer) can reach a lethal burden^23^. We also highlight the 10^9^ threshold because it is a common level for the clinical detection of solid tumors (≈1.2 cm in diameter)^24^; although the detection of smaller neoplasms, in the order of 10^7^ cells, is also possible^23^. The detection threshold is important because even if theoretically replication limits might prevent a tumor from reaching a lethal burden, the mere finding of a neoplasm can significantly impact the life and treatment decisions of patients.

Results in Fig. 2A indicate that (in the range of *k*-values considered) 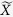 will only reach significant levels if there is a large deviation from the homeostatic condition *r* = 1. In particular, events that produce even moderate changes in the death-to-birth ratio result in relatively small values of 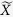. For example, for *k* = 60, 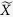 will not reach the detection threshold unless the birth rate equals at least 260% of the death rate (*r* = 0.385). Furthermore, the parameter region that produces a lethal burden is small: the replication capacity of the original cell must be greater than 40 and the imbalance between birth and death very high (low death-to-birth ratio). For *k* = 50, 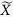 will reach the lethal threshold only when the birth rate is at least 7.7 times the death rate (*r* = 0.13). Overall these results indicate that, based on the maximum size of the expected cell population, replication limits pose a significant barrier to tumor progression if the imbalance between cell death and division is moderate or low. Indeed, the parameter region that leads to values of 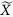 greater than the typical detection threshold is small. Hence, it is possible that a large number of deregulation events that lead to abnormal growth initiate in cells that have a death-to-birth ratio and replication capacity that lead to relatively small maximum cell population sizes. This suggests that replicative limits might halt a significant number of incipient growths before they are ever detected.

We also mention that 40–60 is typically cited as the maximum number of divisions for human fetal cells^1^,^22^. However, some somatic cells might have a higher replication capacity. For example, cells from the BJ cell line (derived from newborn foreskin) are capable of roughly 80 divisions^25^. The replication capacity of adult cells however, is lower and decreases with age^22^. Hence, given that oncogenic events that result in uncontrolled proliferation generally occur later in life, the replication capacity of a cell that initiates abnormal growth is likely to be lower than that of embryonic cells and within the range considered here.

A number of clarifications: First, it is important to distinguish between a ‘high turnover rate’ and ‘high death-to-birth ratio’. The term “high turnover rate” requires that both the death rate and the birth are fast and also that they are nearly balanced. The concept of high death-to-birth ratio on the other hand, says nothing about the absolute speed of the processes of cell division and death. Second, a high turnover rate is often associated with a bad prognosis at the time of diagnosis^26^ because by the time a tumor with a high death-to-birth ratio is detected it will have undergone more divisions, and thus potentially may have acquired more mutations, than a tumor with the same size, which came about as the result of a process with a lower death-to-birth ratio^26^. That is, if we consider two equally sized tumors, one with a high death-to-birth ratio and one with a low death-to-birth ratio, the tumor with the high death-to-birth ratio will have undergone more cell divisions than the tumor with a low death-to-birth ratio. From the point of view of replicative limits we look at a different problem. Given the same replication capacity, a tumor with a high death-to birth ratio compared to a tumor with a low death-to-birth ratio will have: 1) A lower probability of reaching any given size and, 2) on average, a smaller overall number of cell divisions during the entire process, which results in a lower probability of escaping replicative limits (discussed in the next sections). Finally, the fact that telomere length decreases with age might suggest that there is a statistically higher risk of cancer development from abnormal growths that initiate at a young age versus those that initiate in older patients; however, there is also a host of metabolic changes associated with aging, which lie outside the scope of this article and that affect the risk of carcinogenesis at different stages of life^27^.

### Hybrid stochastic-deterministic algorithm

We are interested in populations that originate from a single cell. When the number of cells is small, stochastic fluctuations can have significant effects that propagate during the entire population lifetime. Hence, fundamental quantities, such as the maximum number of cells, are intrinsically stochastic. We need then to evaluate the variability of the outcomes. In particular we want to investigate how representative the expected cell population is and determine the probability that the maximum cell number exceeds the lethal and detection thresholds. We also want to investigate the probability of escaping replication limits, which, as we will later discuss, depends on the distribution of the total number of divisions.

One way to study these questions is to perform computer simulations. An inherent difficulty with this approach is that the size of a population can vary widely during its lifetime (from a single cell at the begining to eventually 10^12^ cells or more). Algorithms that accurately capture the important variability that occurs at the start of a simulation are generally ill-equipped to deal with the very large number of cells that might be present at later stages (making them computationally unfeasible^28^). On the other hand, when the number of cells is large, the population dynamics are well described by the average behavior of the cell population. Therefore, we propose a hybrid stochastic-deterministic algorithm to compute the relevant distributions.

At the start of a simulation, there is a single cell with parameters (*k*, *q*) and the time evolution of the system is determined using a version of the Stochastic Simulation Algorithm^28^. More precisely, let *x*_*ρ*_(*t*) be the number of cells with replication capacity *ρ* at time *t*, and *λ* = *q*∑_*ρ*>0_*x*_*ρ*_(*t*) + (1 − *q*)∑_*ρ*≥0_*x*_*ρ*_(*t*), then the probability that the next event is cell division is *P* = *q*∑_*ρ*>0_*x*_*ρ*_(*t*)/*λ* and the probability that it is cell death is 1 − *P*. If a division event takes place, the probability that it occurs in a cell with replication capacity *j* > 0 is *x*_*j*_(*t*)/∑_*ρ*>0_*x*_*ρ*_(*t*). Similarly, if the next event is cell death, the probability that it occurs in a cell with replication capacity *j* ≥ 0 is *x*_*j*_(*t*)/∑_*ρ*≥0_*x*_*ρ*_(*t*). The time of the next reaction is updated as *t* + *τ*, where *τ* is an exponentially distributed random number with mean 1/*λ*. The stochastic simulation then proceeds until the first of two things occur: 1) the population becomes extinct (or entirely senescent if *q* = 1), or 2) the total number of proliferating cells (*ρ* > 0) reaches a fixed large number *N*, which signals the transition to the deterministic phase. In the deterministic phase we use the last state of the system obtained during the stochastic phase as the initial conditions for the ode system (1), which is then solved numerically. The entire algorithm is coded in C using routines from GNU Scientific Library.

In Fig. 1C,D we present simulations of the total number of cells and the cumulative number of divisions for the fully stochastic and hybrid algorithms, which suggest excellent agreement between the two methods. The parameters selected in these figures (*k* = 40, *q* = 0.75) are such that the implementation of the fully stochastic algorithm through the entire lifetime of the population is computationally feasible. In these and all figures the transition threshold *N* = 10^6^.

### Distribution of the maximum number of cells

In a population that originates from a single cell with replication capacity *k*, the maximum number of cells can take any value between one and 2^*k*^. It is important then to study not only the expected value, but also the distribution of the maximum number of cells. We can estimate this distribution from independent random simulations performed using the hybrid algorithm.

**Figure 3:**
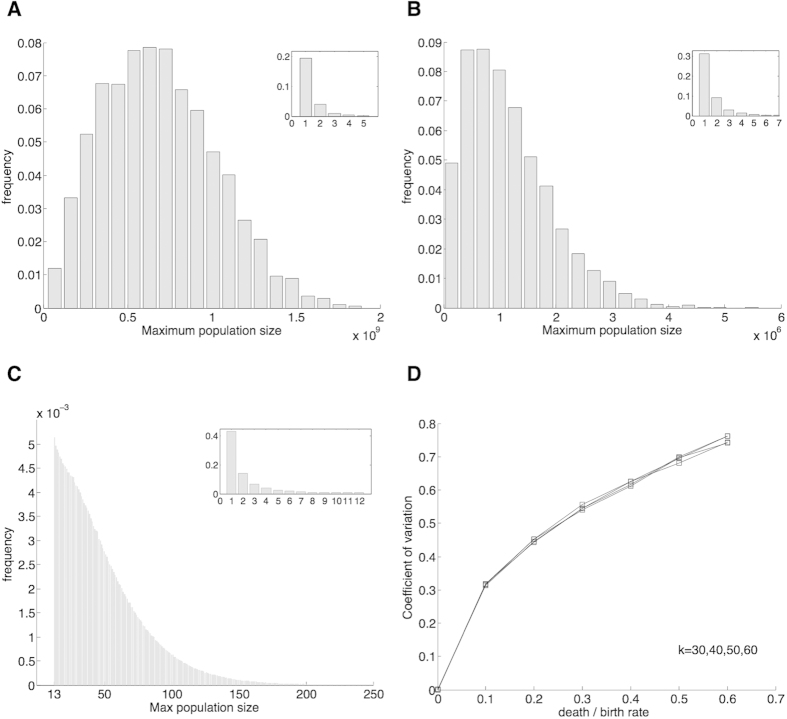
In panels (A,B) the distributions of the maximum population size are bimodal. In (**A**) the maximum number of cells was either more than 2.2 × 10^7^ (major axes, 75% of the time) or less than 5 (inset, 25% of the time); parameters *k* = 45 and *r* = 0.25. In (**B**), which uses parameters *k* = 45 and *r* = 0.45, 99.5% of the time the maximum number of cells was either more than 50,000 (major axes, 53.8%) or less than 8 (inset, 45.7%). In (**C**) the distribution is unimodal (a decreasing function of the maximum number of cells); parameters *k* = 21 and *r* = 0.75. In (**C**) the graph is broken down in two parts (major axes and inset) to present a greater resolution. The frequency of times that the maximum number of cells 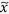 equaled 12 (last value in the inset) is more than the frequency of times that 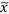 equaled 13 (first value in the major axes). (**D**) Coefficient of variation for samples in which the cell population reaches a large size. Number of simulations: 10^4^ in panels (**A,B,D**); 10^7^ in panel (**C**).

We first note that depending on the parameters, the distribution of the maximum number of cells can be either bimodal or unimodal, where we defined these terms based on the number of local maxima of the probability density function (Fig. 3A–C). In regions where the distribution is clearly bimodal, we typically observed two types of outcomes: either the population was extinguished very early on (never surpassing a small number of cells), or it persisted for much longer periods of time reaching a large size. For example, in 10,000 simulations used to produce Fig. 3A, which uses parameters *k* = 45, and *q* = 0.85, the maximum number of cells was either less than 15 (17% of the time) or more than 10^7^ (83% of the time). Figure 3C depicts a unimodal distribution. In general we found unimodal distributions when the average maximum number of cells is small.

**Figure 4:**
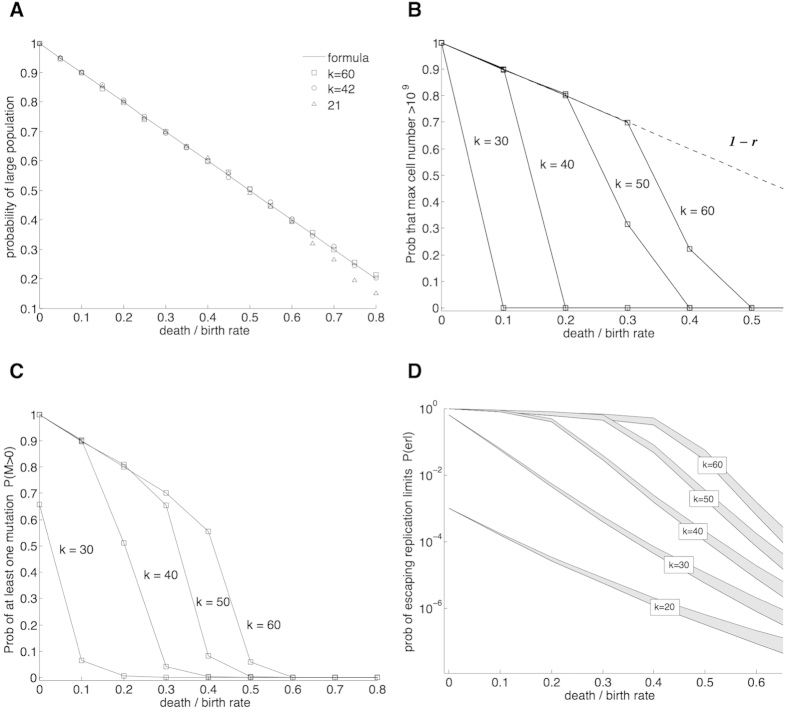
(**A**) Probabilities of reaching a large population (depicted by symbols) as a function of the death-to-birth ratio (*r*) for several values of the replication capacity *k*. A solid line plots the approximation (1 − *r*). (**B**) Probability that the maximum number of cells is greater than 10^9^ (for most values of *r* the probability of reaching 10^9^ cells is either zero or very close to the approximate probability of reaching a large size (1 − *r*)). (**C**) Probability that at least one mutation takes place P(*M* > 0). (**D**) Probability of escaping replication limits through a mutation that activates telomerase P(erl). For each of the values of *k*, P(erl) falls inside the corresponding grey band. The lines that form the boundary of the bands are calculated using the upper and lower bounds in Eq. 9. In (**C**,**D**) the mutation rate per cell division *μ* = 10^−9^. Results based on 10^4^ simulations per point.

We want to focus on parameters that produce on average at least a moderate maximum cell population (defined here as ≥10^6^). This requirement defines region *A* (Fig. 2B). In *A* distributions are bimodal: For each parameter sampled, in at least 99.5% of the simulations the maximum number of cells was either less than 20 or more than 50,000. In this region the probability that a population reaches a large size can be approximated as the probability of becoming infinitely rich in the classic Gambler’s Ruin problem, which equals 1 − *r*^19^. For *k* ≥ 20 this formula provides an excellent approximation (Fig. 4A).

For the rest of this section we focus on region *A*, which has the following properties: 1) the expected maximum population size is not very small (≥10^6^), 2) the distributions are clearly bimodal, and 3) the probability of reaching a large size is accurately approximated by 1 − *r*. Distributions for parameters that are close to A have similar properties. Further away from *A* the population sizes become smaller and distributions are unimodal. In these regions the maximum cell population is by definition small, several orders of magnitude below the detection threshold, and unlikely to affect the health of an individual.

We want to study the variation in *A* around the two modes. First, if the cell population is extinguished early on, the maximum number of cells is close to zero. To study the variability around the larger mode we compute the coefficient of variation (defined as the sample deviation over the sample mean), conditioning over those samples where the cell population reaches a large size. The coefficient of variation (CV) provides a normalized measure of dispersion for the probability distribution. Figure 3D plots CV as a function *r* for different values of *k*. This figure suggests that if rapid extinction does not occur, there is relatively low variability in the maximum number of cells. Indeed, in the sample distributions when the population reaches a large size, the maximum number of cells fell within one order of magnitude of the mean at least 96% of the time. Moreover, in all instances the ratio of the 95th to the 5th percentile was always less than 20.

These results indicate that if we are primarily concerned with the order of magnitude of the maximum number of cells, then in *A* the distribution of the maximum number of cells is well characterized by two numbers: (1) the probability of reaching a large population size (≈1 − *r*), and (2) the expected value of the maximum number of cells if a large population size is reached. Figure 4B exemplifies this characterization: For most parameter values the probability of reaching the detection threshold (10^9^) is either very close to the probability of reaching a large size (if the second mode is large enough), or zero otherwise. Figure 2C exemplifies this same idea. The probability of reaching the detection threshold defines clearly two regions in the parameter plane. In one region the probability of reaching this threshold is ≈1 − *r* and in the other it is essentially zero. In the boundary of these two regions the large modes of the distributions lie close to the detection threshold. The relatively sharp transition observed at the boundary is indicative of the previously discussed low variability of the distributions.

Finally, we discuss how the maximum value of the expected cell population 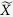 relates to the probability of reaching a particular threshold. First, let 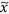 be the maximum population size for a given realization of the process (in general 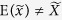). Following this notation let 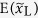 be the expected maximum cell population conditioning over the samples where the population reaches a large size. We find that in *A*, 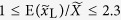. We previously found that in this region 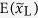 and 1 − *r* approximately determine the probability of reaching a particular threshold. Hence, given that 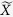 and 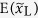 have the same order of magnitude, the value of 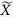 should provide a good estimate of the region where the probability of reaching a given threshold is not negligible. In Fig. 2C we verify this directly for the detection threshold; here, the region where 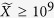 (dashed line) provides an excellent approximation to the region where the sample probability of reaching this threshold is greater than zero. More generally we can say that in *A* the values of 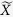 computed using Eq. 7 gives us a good estimate of the region where the probability of reaching a particular threshold is significant.

### Probability of escaping replication limits

Telomerase is a RNA/protein complex that adds telomeric DNA to the ends of linear chromosomes. And there is also evidence which suggests that different components of the telomerase enzyme may promote cell proliferation and survival by non-canonical functions^29^,^30^. Embryonic stem cells and germ line cells escape replicative limits by expressing telomerase at high levels^2^. Certain kinds of adult stem cells also express telomerase albeit at lower levels, which allows them to divide a large yet still limited number of times^31^, whereas other types of normal cells do not express it. Most cancers escape replicative limits also by expressing telomerase (90%)^21^ and less frequently through the alternative telomere lengthening (ALT) pathway (10%)^32^.

Even if adult stem cells do not express sufficient telomerase to maintain a stable telomere length, they possess a replication capacity that far exceeds that of non-stem cells. For example, colon stem cells are estimated to divide more than 5000 times in a lifespan of 80 years and hematopoietic stem cells roughly 960 times during the same time period^33^. Hence, in tumors that initiate in telomerase positive stem cells replicative senescence is unlikely to act as a significant barrier to tumor progression^19^. In telomerase negative cells the escape from replicative limits can occur through a mutation that activates telomerase. Recently frequent somatic mutations that activate the core promoter of telomerase have been identified in multiple types of tumors including, amongst others, melanomas (71%), hepatocellular carcinomas (59%) and gliomas (51%)^34^,^35^,^36^. In this section we will study the probability that a cell population escapes replication limits through a mutation that activates telomerase.

First, let us extend the definition of *ρ* (previously defined as the replication capacity of a cell) and think of it as a division counter. As before the cell population starts with a single cell with a given counter *ρ* = *k*. When a cell with counter *ρ* divides the two daughter cells that result from the division will have a counter equal to *ρ* − 1. If a cell has not activated telomerase, then cell division stops once the counter hits *ρ* = 0. On the other hand, if telomerase has been activated, the cell can keep on dividing past the zero mark and the counters of its progeny can progress into negative integers (*ρ* = −1, −2, …).

If we assume that mutations take place during cell division, then the probability of acquiring a mutation is directly related to the total number of divisions that occur in cells with counters *ρ* > 0. Indeed, given a realization of the process, let *D* be the total number of divisions that took place in cells with *ρ* > 0. Then, if *μ* is the mutation rate of telomerase activation per cell division, the probability that no mutation took place is *P*_0_ = (1 − *μ*)^*D*^. Note that *D* is a random variable that depends on the parameters (*k*, *q*) of the original cell. Hence, using conditional probabilities it is straightforward to see that the probability that at least one mutation takes place is:


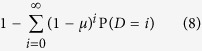


We now introduce some notation. We will write erl as an abbreviation for the phrase “escaping replication limits” and call *M* the total number of mutations that take place (note that the value of P(*M* > 0) is given by Eq. 8). We have then: P(erl) = ∑_*i*≥0_P(erl|*M* = *i*)P(*M* = *i*). Because the P(erl|*M* = 0) = 0 it follows that P(erl) ≤ P(*M* > 0).

The cell population will escape replication limits only if the progeny of at least one mutant does not stochastically die out. Given that mutants are unconstrained by replication limits the number of cells originating from a single mutant can be seen as a random walk in the non-negative integers. The probability that this random walk never reaches the absorbing state (zero cells) is 1 − *r*^19^. It follows that if *i* mutations take place, then the probability that the progeny of all the original mutants die out is *r*^*i*^ and thus P(erl|*M* = *i*) = 1 − *r*^*i*^. Since *r* ≤ 1 we have 1 − *r* ≤ 1 − *r*^*i*^ for all *i* > 0 and it follows that ∑_*i*>0_(1 − *r*)*P* (*M* = *i*) ≤ P(erl). Combining this relationship with the previously found inequality we find an upper and lower bound for P(erl):


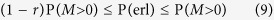


Given a set of parameters (*k*, *q*) consider two types of processes: in the first type the activation of telomerase is a possibility and in the second type it is not. The distribution of the total number of divisions by cells with counter *ρ* > 0 in the first type of process is exactly the same as the distribution of the total number of divisions in the second type. Hence, we can use the hybrid algorithm to calculate the probabilities in Eq. 8 and obtain numerically the value of P(*M* > 0). In a given simulation if *D*_S_ is the total number of divisions that occur during the stochastic phase of the algorithm and [*t*_1_, *t*_2_] is the time interval where the deterministic phase takes place, then the total number of divisions is 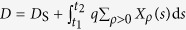. In Fig. 4C we plot *P*(*M* > 0) for several values of the replication capacity *k* using the approximate point mutation rate in cancer of 10^−9^ for *μ*^37^.

The upper and lower bounds in Eq. 9 define a band of width *r*P(*M* > 0) where P(erl) lies (Fig. 4D). The relative widths of these bands (*r*) decreases as the death-to-birth ratio *r* becomes smaller. In Fig. 2D we present a heat map of the probability of escaping replication limits where we estimate the value of P(erl) as the midpoint between the upper and lower limits.

We find that moderate replication capacities and imbalances of the death-to-birth ratio result in substantial protection against cancer. For example for *μ* = 10^−9^, if *k* ≤ 35 and *r* > 0.5, then P(erl) is less than one in one thousand. What is also clear however, is that the probability of escaping replication limits through the activation of telomerase considerably expands the parameter region in which abnormal cell proliferation poses a significant risk. Indeed, a comparison of Fig. 2A,B demonstrates that populations, which based on their replication limits should be destined to remain at an innocuous microscopic level, have a non-negligible probability of activating telomerase. Moreover, parameters that produce on average a maximum number of cells close to 10^9^ (adding to a tumor volume that by itself is unlikely to be harmful) result in a probability of escaping replication limits that is close to one.

One important biological question is whether cancers typically originate in cells that are already telomerase positive (such as stem cells), or telomerase can be acquired later, after uncontrolled proliferation begins. The fact that telomerase is detected in approximately 90% of human cancers could suggest that cancer originates in cells that express telomerase^21^. According to our model however, if a cell population surpasses the typical detection size of 10^9^ cells, there is a very high probability that telomerase will be activated (Fig. 2B). Hence, even if the abnormal growth originates in a telomerase negative cell, there is a very high probability that the cell population will be expressing telomerase by the time the abnormal growth is found.

### Proposed applications to anti-telomerase cancer therapy

Telomeres and telomerase have long been identified as potential targets for cancer therapy^38^. In particular, the inhibition of telomerase aimed at triggering telomere-initiated senescence or apoptosis has garnered considerable attention as a possible therapeutic strategy. One telomerase inhibitor, Imetelstat (GRN163L), is currently on phase II clinical trials^39^. A potential problem with telomerase inhibitors is that they require a lag time (several cell divisions) before telomeres shorten to a critical length^40^. Indeed, if the replication capacity of cancer cells before telomerase is inhibited is large enough, the tumor might progress to lethal levels before the effects of telomerase inhibition are observed. In this context a quantitative understanding of the dynamics of a cell population subject to replicative limits can provide crucial insights into the effectiveness and applicability of therapies aimed at inducing telomere-mediated senescence in cancer cells. A crucial question is, how much will the number of cancer cells increase during the lag-time between the time when telomerase inhibition starts and the time when senescence or apoptosis occurs?

Next we propose an application on how the model could be potentially used to estimate the increase in cancer cells that occurs post-telomerase inhibition, based on patient-specific parameters. These guidelines provide a starting point to use insights from quantitative methods to predict the outcomes of anti-telomerase therapies. Ultimately however, the success of this approach can only be determined through validation with experimental data obtained as part of a collaborative process.

First, the distribution of the lengths of the shortest telomeres in cancer cells of an individual patient should be measured. This distribution can be used to estimate the replication capacity of the cell population. Note that the length of the shortest telomeres in a cell should be measured and not just the average telomere length in the cell, as senescence depends not on average telomere length but on the length of the shortest or few shortest telomeres^41^. (Methods that measure the length of individual telomeres, e.g. Q-FISH, can determine the length of the shortest telomeres, but may lack the resolution to determine with confidence which one exactly is the very shortest one.) While the length of the shortest telomere, at which a cell will senesce or become apoptotic is context dependent^14^, the average length at which this occurs can be experimentally determined in specific cancers and used as a baseline for patients. Similarly, the rates of cell division and death can be measured in individuals or the averages for a specific type of cancer can be used as an estimate. Once these parameters are established, the application to the model would be straightforward. To calculate for example the maximum size of the expected cell population 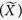 post-treatment, the distribution of telomere length and the current tumor size could be used to obtain the initial conditions in Eq. 6; here, if the time at which measurements are taken is set as *t* = 0, the number of cells with replication capacity *j* at the time of the measurement is *X*_*j*_(0). The hypothesis that telomere length can influence the success of anti-telomerase therapy is suggested by preliminary findings from a phase II study of Imetelstat as maintenance therapy for non-small-cell lung cancer, which showed a trend towards improvement in progression-free survival and overall survival in patients with short telomere length^39^.

It has been proposed that anti-telomerase therapeutics should be used in conjunction with other forms of cancer treatment, for example chemotherapy. In this case, the model could potentially also be used to estimate the outcomes. For example, if telomerase inhibitors are given at the same time as chemotherapy treatments, the effects of chemotherapy could be modeled as an increase in the death-to-birth ratio *r* and thus, the effect of combining both treatments can be estimated quantitatively.

Mouse models have shown that anti-telomerase therapy can lead to alternative lengthening of telomeres (ALT), which allows cancer to survive and spread independent of telomerase^42^. Detailed models of ALT require knowledge of the number and nature of the events that drive its emergence, which at the current moment are poorly understood^32^. However, in the context of a cancer cell population in which telomerase has been inhibited, as a first step, the emergence of ALT can be modeled non-mechanistically as a random event in cells restricted by replicative limits. In this case, the procedure used to estimate P(erl) would also provide an estimate for the probability of developing resistance to telomerase inhibitors. Similarly, if ALT is activated as a two-step process, the model can be adapted by using the probability generating function for two mutations in a birth-death process^43^, and then applying the methods in the generalized Luria-Delbrück model^44^.

Finally, in this article we consider the canonical function of the telomerase complex in extending telomere length and how inhibition of this canonical function can affect a cancer cell population. However, blocking telomere elongation might not be the only way that telomerase inhibition may be exploited for cancer therapy. In particular, *in vitro* studies in breast and pancreatic cancer cell lines suggest that imetelstat treatment may also deplete cancer stem cells through a mechanism independent of telomere shortening^45^.

## Discussion

Multiple lines of evidence support the role of replicative senescence as a tumor suppressor pathway. First, significant telomere attrition has been found in multiple types of precancerous tissues^2^. There is also substantial evidence to associate cellular senescence with pre-malignant stages of tumor development. In humans for example, senescent cells have been identified in benign lesions of the skin, neurofibromas, and benign lesions of the prostate^3^. In mice the analysis of tumors initiated by the endogenous expression of the oncogene ras showed an abundance of senescent cells in premalignant lesions of the lung, but no signs of cellular senescence in lung adenocarcinomas^3^. Moreover, frequent somatic mutations that activate telomerase have been identified in various cancers^34^,^35^,^36^. Together these findings suggest that replicative senescence restricts tumor progression. However, direct evidence that most precancerous cells senesce remains elusive.

Our analysis suggests that, if most deregulation events produce at most moderate imbalances between cell birth and death, then replicative senescence is likely to suppress tumor progression before any abnormalities are clinically diagnosed. Alternatively, if oncogenic events lead to low death-to-birth ratios, then the risk of cancer development can be significant, especially through the probability of activating telomerase. Low death-to-birth ratios can occur for example, if excessive cell division is caused by abnormal stimuli in tissues with normally low cell turnover rates. These findings underscore the necessity of understanding not only the net growth rate of tumors, but also the rates of cell division and death in specific tumors. They also highlight the importance of coadjuvant factors that promote cell death as part of the replicative senescence tumor suppressor pathway. Indeed, replicative senescence might be efficient as a cancer protecting mechanism when it works in tandem with other factors that contribute to high rates of mortality in tumor cells. Such factors have been documented in numerous types of cancers. For example, poor neovascularization can contribute to high levels of apoptosis at early stages of tumor development^46^. The immune system also contributes to the clearance of abnormal cells. Significantly, senescence surveillance of premalignant cells by the immune system has been shown to be an important component of the senescence anti-tumor barrier in hepatocellular carcinomas^47^. The same ideas apply to anti-telomerase therapeutics. As we discussed, the response to telomerase inhibitors applied in conjunction with chemotherapy will depend on the increase in the death-to-birth ratio of cancer cells upon chemotherapy treatment.

We also found that by the time tumors reach a size where they are typically detected, there is a high probability that telomerase is activated in the cell population, even if the cell of origin was telomerase negative. In this regard we note that factors independent of replicative limits can influence the likelihood of tumor initiation in different cell types, such as the spatial location of stem cells and non stem cells in specific tissues (e.g. the spatial dynamics in the colon crypt favor a stem cell origin in colon cancer^48^). What is also clear is that even moderate extensions of the replication capacity, beyond the maximum of 60 divisions used in this article, can significantly diminish the potential of replicative senescence as a tumor suppressor mechanism. Hence, replication limits might be ineffective not only in stem cells, but also in cells with extended, yet still limited proliferative potential such as progenitors.

To finalize we discuss possible adaptations of the model. First, in the stochastic phase of the hybrid algorithm, different distributions other than exponential can be used to model the events of cell division and cell death. The corresponding expected rates for each of these events can then be used in the deterministic phase of the algorithm. Second, multiple factors such as competition for space and oxygen, and the need for angiogenesis can affect the growth rate of cancer at different stages, which can result in a deceleration of growth as the size of the tumor increases, coupling the rates of cell division to the size of the tumor. Hence, in the model the rates of cell division and death can be made to depend on the size of the cell population as to correspond with different sigmoidal tumor growth laws^49^. In a separate, but related issue, when considering the aging process, the rates of cell death might increase with age. Finally, cancers can carry mutations (e.g. in PARP1 and BRCA1) that affect the DNA repair machinery and can increase the mutation rates^20^. Hence, a possible extension of the model is the inclusion of an additional mutation that increases the mutation rate. These and other adaptations of the model are the subject of future research.

## Additional Information

**How to cite this article**: Rodriguez-Brenes, I. A. *et al*. Quantifying replicative senescence as a tumor suppressor pathway and a target for cancer therapy. *Sci. Rep*. **5**, 17660; doi: 10.1038/srep17660 (2015).
